# Die Bedeutung der Regionalisierung für die Gesundheit

**DOI:** 10.1007/s00103-025-04127-0

**Published:** 2025-09-24

**Authors:** Jobst Augustin, Uwe Koch-Gromus

**Affiliations:** 1 Institut für Versorgungsforschung in der Dermatologie und bei Pflegeberufen (IVDP), Universitätsklinikum Hamburg-Eppendorf (UKE), Martinistraße 52, 20246 Hamburg, Deutschland; 2 Institut und Poliklinik für Medizinische Psychologie, Universitätsklinikum Hamburg-Eppendorf, Martinistr. 52, 20246 Hamburg, Deutschland

Als wissenschaftliche Disziplin befasst sich die Geografie mit der Erfassung, Beschreibung und Analyse von natürlichen und menschengemachten räumlichen Strukturen, Prozessen und Wechselwirkungen sowie Orten des menschlichen Lebens und Handelns. Die Gesundheitsgeografie setzt sich als Teildisziplin mit raumbezogenen gesundheitsspezifischen Fragestellungen auseinander und verfolgt dabei einen holistischen Ansatz, das heißt eine ganzheitliche Betrachtung von Raum, Umwelt, Gesellschaft und Gesundheit.

Die COVID-19-Pandemie hat gezeigt, wie bedeutsam der Raum für gesundheitsspezifische Fragestellungen ist. Das betrifft sowohl die räumliche Dynamik der Virusverbreitung als auch sozialräumliche Aspekte, etwa die Inanspruchnahme von Impfungen. Die Analyse räumlicher gesundheitsspezifischer Sachverhalte erfordert zumeist umfangreiche (Geo‑)Datensätze und entsprechende Methoden zur Analyse (z. B. Techniken der Verschneidung von Geo- und Gesundheitsdaten, Verfahren der räumlichen Statistik) und Visualisierung. Damit gehen zahlreiche Herausforderungen einher, beispielsweise bei der Datenverarbeitung. Gute wissenschaftliche Praktiken (z. B. *Gute Praxis Erreichbarkeitsanalysen im Gesundheitswesen*) im Feld der Gesundheitsgeografie dienen dazu, die Qualität der Verarbeitung, Analyse und Visualisierung von Daten zu gewährleisten.

Ein zentraler Fokus der Gesundheitsgeografie liegt auf den Ursachen räumlicher Variationen von Gesundheit und Krankheit. So stellt sich beispielsweis die Frage, ob eine räumliche Assoziation zwischen Mortalität und Früherkennung bei Krebserkrankungen existiert. Auch die soziale Lage spielt eine wichtige Rolle bei der Erklärung räumlicher Variationen, beispielsweise im Hinblick auf das Auftreten psychischer Erkrankungen.

Eng daran geknüpft ist die regionsspezifische Betrachtung der Gesundheitsversorgung, denn markante räumliche Variationen finden sich auch in der Gesundheitsversorgung wieder. Ein bislang in diesem Zusammenhang wenig diskutiertes Thema ist die Palliativversorgung in Deutschland, die beispielhaft für regionale Unterschiede dienen kann. Auch die Inanspruchnahme präventiver Gesundheitsmaßnahmen, wie beispielsweise Impfungen, ist aus räumlicher Perspektive von hoher Relevanz, da sich auch hier markante (unerwünschte) regionale Unterschiede zeigen.

Die physische Umwelt kann sowohl negative Effekte auf die Gesundheit haben (z. B. Luftschadstoffe als Risikofaktor für Atemwegserkrankungen) als auch positive (z. B. Steigerung des Wohlbefindens durch das Erleben naturnaher Landschaften). Die Exposition gegenüber Risikofaktoren und die Möglichkeit der Teilhabe an einer naturnahen Umwelt hängen dabei vom Wohnort, der sozialen Lage und weiteren Faktoren ab.

Dieses Themenheft gibt anhand ausgewählter aktueller Fragestellungen einen Einblick in die gesundheitsgeografische Forschung. Die Beiträge konzentrieren sich dabei auf die theoretischen und methodischen Grundlagen der Gesundheitsgeografie, auf räumliche Variation von Krankheit und Gesundheit sowie auf die regionale Versorgungsforschung.

Die ersten beiden Beiträge befassten sich mit Grundlagenfragen des Themenheftes „Geografie und Gesundheit“. Zunächst geben *C. Buck et al*. einen Einblick in die Gesundheitsgeografie anhand des Beispiels der COVID-19-Pandemie. Die Ergebnisse ihrer Analysen an Daten der COVID-19-Pandemie in Bremen zeigen eindrucksvolle räumliche Unterschiede in den COVID-19-Fallzahlen und mehr Fälle in deprivierten Ortsteilen. Die Studie belegt nach Einschätzung der Autor:innengruppe, dass der gewählte Ansatz zur Identifizierung vulnerabler Bevölkerungsgruppen geeignet ist und eine gezielte Implementierung von Präventionsmaßnahmen erlaubt. Im zweiten Grundlagenbeitrag setzen sich *E. Swart et al*. mit der Bedeutung raumbezogener Gesundheitsdaten und wissenschaftlichen Standards im Rahmen der Gesundheitsgeografie auseinander. Sie verweisen darauf, dass eine angemessene Nutzung potenziell geeigneter Daten deutlich von den in der Studie verfolgten Zielsetzungen und methodischen Fragestellungen abhängt.

Der nachfolgende Beitrag von *L. Wollgast et al*. prüft Zusammenhänge von regionaler sozioökonomischer Deprivation sowie von sozioökonomischem Status mit depressiver Symptomatik. Die Analyse der Daten der Studie Gesundheit in Deutschland aktuell (GEDA) (2019/2020) zeigt – entgegen der Annahme der Autor:innen – keine systematische geografische Verbreitung der Depressivität über Deutschlands Stadt- und Landkreise. Das Depressionsrisiko ist allerdings in sozioökonomisch hoch bzw. mittel deprivierten Gemeinden weitaus höher als in niedrig deprivierten Gemeinden.

Die folgenden beiden Beiträge befassen sich mit regionalen Unterschieden in der Gestaltung unterschiedlicher Versorgungsangebote. Zunächst untersuchen *T. Petzold et al. *die Bedeutung der Regionalität am Beispiel der Verfügbarkeit eines wohnortnahen Zugangs zur stationären Palliativversorgung. Mit einer spezifischen Analysetechnik (E2SFCAM) zeigen die Autor:innen, dass der Zugang zu Palliativstationen in Deutschland stark zwischen und innerhalb von städtischen und ländlichen Gebieten variiert. *M.K. Akmatov et al.* untersuchen anschließend räumliche Unterschiede und regionale Einflussfaktoren der Influenza-Impfquote bei Personen ab 60 Jahren auf der Basis von vertragsärztlichen Abrechnungsdaten. Die komplexen Datenanalysen weisen auf eine erhebliche Variation der Influenza-Impfquote auf Kreisebene zwischen 10 % und 61 % hin und erlauben die Identifizierung von räumlichen Clustern und von Risikofaktoren für niedrige Impfquoten. Daraus lassen sich regional zugeschnittene Interventionsmaßnahmen zur Verbesserung der Impfinanspruchnahme ableiten.

*J. Augustin et al.* gibt eine Übersicht über Maßnahmen zur Sicherstellung der ambulanten Versorgung in ländlichen und strukturschwachen Regionen. Die hier zur Anwendung kommenden vielfältigen konzeptionellen Ansätze und Maßnahmen reichen von der Verbesserung der Kontextgestaltung (z. B. Niederlassungsförderung durch Zurverfügungstellung günstigen Baulands) bis hin zur Implementierung telemedizinischer Maßnahmen. Der Evaluation solcher Maßnahmen und deren Regionsspezifität kommt in diesem Kontext besondere Bedeutung zu.

Die nachfolgenden 3 Beiträge beziehen sich auf das Thema Umwelt und Gesundheit**. **Die Untersuchung von *S. Hischke et al. *zeigt in Hamburg im Zentrum besonders erhöhte Luftschadstoffbelastungen von Feinstaub auf. Auch konnte eine positive Assoziation von Feinstaubkonzentrationen und Erkrankungen sowohl des Atemwegs- als auch des Herz-Kreislauf-Systems aufgezeigt werden. Die Ergebnisse liefern Ansatzpunkte für gezielte Maßnahmen zur Senkung des Erkrankungsrisikos. Der sich anschließende Beitrag von *T. Kistemann und T. Falkenberg *beschreibt und analysiert ideengeschichtlich und auf der Grundlage unterschiedlicher theoretischer Ansätze das Konzept der *„*therapeutischen Landschaften“. Dies bietet einen analytischen Rahmen zum besseren Verständnis der Bedeutung von spezifischen Orten und Landschaften für Gesundheit und gesundheitliches Wohlbefinden. *G. Czwikla et al*. setzen sich im letzten Beitrag dieses Themenblocks mit der potenziellen Bedeutung räumlicher Analysen für das Bestreben der Gewährleistung von Umweltgerechtigkeit auseinander. Hier sehen die Autor:innen derzeit aber noch eindeutige Grenzen in der Umsetzung. Um begründete politische Entscheidungen treffen zu können und evidenzbasiert eine Reduktion sozial ungleich verteilter Umweltbelastungen und -ressourcen sowie deren gesundheitlicher Effekte zu erreichen, bedarf es einer deutlichen Verbesserung der Datenlage als Voraussetzung für ein umfassendes, einheitliches Monitoring.

Der letzte (englischsprachige) Beitrag des Themenheftes von *G. McCartney und D. Walsh* behandelt das Rahmenthema aus einer international vergleichenden Perspektive. Die Autoren analysieren die seit 2010 im Vereinigten Königreich, in Deutschland und anderen Ländern mit hohem Einkommen zu beobachtende deutliche Verlangsamung des Anstiegs der Lebenserwartung und des Rückgangs der Sterblichkeit. Diese erklären sie aufgrund von Daten aus verschiedenen Landesteilen (z. B. Schottland und England) im Wesentlichen durch eine deutliche Verschlechterung der Sterblichkeitsraten in ärmeren Bevölkerungsgruppen. Als mögliche Ursachen sehen die Autoren wirtschaftliche „Sparmaßnahmen“, die nach der Finanzkrise von 2007/2008 umgesetzt wurden.

Liebe Leserinnen und liebe Leser, wir hoffen, dass es durch die Auswahl der Beiträge des Themenheftes gelungen ist, ein informatives Bild der aktuellen Forschung und Diskussion zum Thema „Geografie und Gesundheit“ zu vermitteln. Wir danken allen Autorinnen und Autoren der Beiträge und den Mitarbeiterinnen der Redaktion des Bundesgesundheitsblattes für die sehr gute Zusammenarbeit und wünschen Ihnen, liebe Leserinnen und Leser, eine anregende Lektüre.

